# Mitochondrial respiratory chain function promotes extracellular matrix integrity in cartilage

**DOI:** 10.1016/j.jbc.2021.101224

**Published:** 2021-09-22

**Authors:** Kristina Bubb, Tatjana Holzer, Janica L. Nolte, Marcus Krüger, Richard Wilson, Ursula Schlötzer-Schrehardt, Jürgen Brinckmann, Janine Altmüller, Attila Aszodi, Lutz Fleischhauer, Hauke Clausen-Schaumann, Kristina Probst, Bent Brachvogel

**Affiliations:** 1 Department of Pediatrics and Adolescent Medicine, Experimental Neonatology, Medical Faculty and University Hospital Cologne, University of Cologne, Cologne, Germany; 2 Center for Biochemistry, Medical Faculty and University Hospital Cologne, University of Cologne, Cologne, Germany; 3 Institute of Genetics and Cologne Excellence Cluster on Cellular Stress Responses in Aging-Associated Diseases (CECAD), University of Cologne, Cologne, Germany; 4 Central Science Laboratory, University of Tasmania, Hobart, Tasmania, Australia; 5 Department of Ophthalmology, University Hospital Erlangen, Friedrich-Alexander-University of Erlangen-Nürnberg, Erlangen, Germany; 6 Department of Dermatology, Institute of Virology and Cell Biology, University of Lübeck, Lübeck, Germany; 7 Cologne Center for Genomics, University of Cologne, Cologne, Germany; 8 Berlin Institute of Health at Charité, Core Facility Genomics, Berlin, Germany; 9 Max Delbrück Center for Molecular Medicine in the Helmholtz Association, Berlin, Germany; 10 Department for Orthopaedics and Trauma Surgery, Musculoskeletal University Center Munich (MUM), University Hospital, Ludwig-Maximilians-University (LMU), Munich, Germany; 11 Center for Applied Tissue Engineering and Regenerative Medicine, Munich University of Applied Sciences, Munich, Germany

**Keywords:** extracellular matrix, matrix metalloproteinase, mitochondria, mitochondrial respiratory chain, transcriptomics, matrisome, single-cell RNA-Seq, THBS1, MMP10, atomic force microscopy, AFM, atomic force microscopy, cDNA, complementary DNA, DHLNL, difunctional dihydroxylysinonorleucine, ECM, extracellular matrix, FDR, false discovery rate, HP, hydroxylysylpyridinoline, MATN1, matrilin-1, mtDNA, mitochondrial DNA, mtRC, mitochondrial respiratory chain, PCNA, proliferating cell nuclear antigen, PFE, proximal femoral epiphysis, PH/H, prehypertrophic/hypertrophic, RT, room temperature, scRNA-Seq, single-cell RNA-Seq, SDH, succinate dehydrogenase, THBS1, thrombospondin 1, TW, Twinkle, UMI, unique molecular identifier, VEGF, vascular endothelial growth factor

## Abstract

Energy metabolism and extracellular matrix (ECM) function together orchestrate and maintain tissue organization, but crosstalk between these processes is poorly understood. Here, we used single-cell RNA-Seq (scRNA-Seq) analysis to uncover the importance of the mitochondrial respiratory chain for ECM homeostasis in mature cartilage. This tissue produces large amounts of a specialized ECM to promote skeletal growth during development and maintain mobility throughout life. A combined approach of high-resolution scRNA-Seq, mass spectrometry/matrisome analysis, and atomic force microscopy was applied to mutant mice with cartilage-specific inactivation of respiratory chain function. This genetic inhibition in cartilage results in the expansion of a central area of 1-month-old mouse femur head cartilage, showing disorganized chondrocytes and increased deposition of ECM material. scRNA-Seq analysis identified a cell cluster–specific decrease in mitochondrial DNA–encoded respiratory chain genes and a unique regulation of ECM-related genes in nonarticular chondrocytes. These changes were associated with alterations in ECM composition, a shift in collagen/noncollagen protein content, and an increase of collagen crosslinking and ECM stiffness. These results demonstrate that mitochondrial respiratory chain dysfunction is a key factor that can promote ECM integrity and mechanostability in cartilage and presumably also in many other tissues.

A large amount of energy is required to drive skeletal growth and synthesize the structural components of the cartilage extracellular matrix (ECM) that withstand mechanical forces and maintain lifelong mobility. The mitochondrial respiratory chain (mtRC) is the major source of cellular energy, but the connection between respiratory activity and ECM homeostasis is still poorly understood (1). We recently induced the expression of the mitochondrial (mt) DNA Twinkle (TW) helicase mutant K320E (2, 3) specifically in growth plate cartilage by Col2a1-directed expression of Cre recombinase (Cre; (4, 5)) to generate mice with impaired mtRC function in chondrocytes (CreTW) (Fig. 1*A*). These mice developed postnatal growth retardation and a chondrodysplasia-like phenotype caused by disturbed metabolic signaling and destabilization of the cartilage-to-bone junction during postnatal development (6). Bulk RNA transcriptome profiling showed that lack of respiration was translated into a general integrated stress response, but the individual load of mtRC dysfunction in chondrocyte subpopulations and the impact on ECM homeostasis remained unclear.

**Figure 1:**
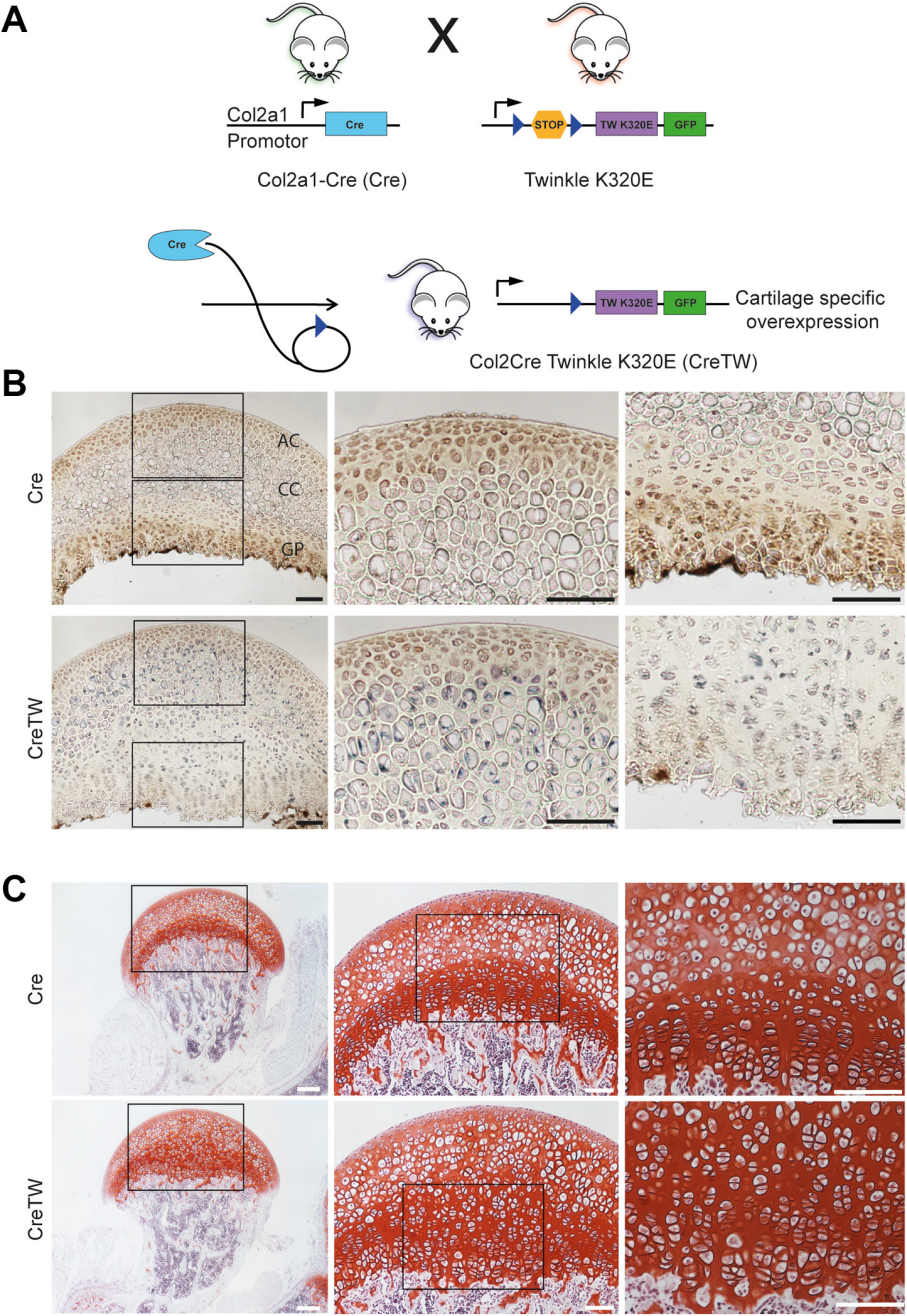
**Characterization of mitochondrial respiratory chain activity and histomorphology in PFE cartilage isolated from 1-month-old Cre or CreTW mice.***A*, generation of mice with a cartilage-specific expression of mutated Twinkle helicase (K320E) using the Cre/loxP system. Mice expressing Cre recombinase driven by the Col2a1 promotor (*blue*, Cre) were crossed with R26-K320E-Twinkle mice to generate offspring with a removal of a stop cassette (*circle*) in the Rosa26 locus to induce the expression of the twinkle mutant K320E (*violet*) and GFP (*green*) only in cartilage (CreTW). Lox site (*triangle*, *dark blue*) (*B*) CYTOCOX (*brown*), and SDH activity (*blue*) activity staining on PFE sections of 1-month-old Cre or CreTW mice. Overview and close-up views of the articular (AC), calcified (CC) (*center*), and growth plate cartilage (GP) (*right*) are shown. Bars represent 100 μm. *C*, overview and close-up views of safranin O–stained PFE sections from 1-month-old Cre or CreTW mice. *Squares* within the overview images represent areas magnified in the close ups. Bars represent 200 μm (*overview*), 100 μm (*close ups*). PFE, proximal femoral epiphysis; SDH, succinate dehydrogenase.

Single-cell RNA-Seq (scRNA-Seq) has evolved as a method to provide a high-resolution map of cellular transcriptomes with the promise of improving the understanding of individual cell function and dysfunction (7). In joint biology, scRNA-Seq was used to study cellular heterogeneity and lineage specification during development. Tissue-specific cell clusters of hind limb development were identified, distinct transcriptome signatures of joint progenitor cells were described (8, 9), and transcriptional profiles of the major developmental paths for joint progenitors were determined (10). scRNA-Seq analysis could also confirm the cluster-specific expression of genes in growth plate cartilage (11) originally defined by oligoarray-transcriptome profiling (12). This technology could therefore be suited to correlate activated ECM-related genetic programs in chondrocyte subpopulations with impaired mtRC activity, but to our knowledge, scRNA-Seq was not yet applied to high-resolution transcriptome analysis of mutant mice with a cartilage phenotype.

The aim of this study was to define the individual cellular load of mtRC dysfunction and define the impact on ECM homeostasis and cartilage stability using a combined approach of high-resolution scRNA-Seq, mass spectrometry/matrisome analysis, and atomic force microscopy (AFM) in mutant mice with cartilage-specific inactivation of mtRC function.

## Results

The mouse proximal femoral epiphysis (PFE) is a unique source of cartilage to isolate sufficient cell numbers for scRNA-Seq analysis. We wanted to analyze older mutant mice with increased mtRC deficiency and therefore first determined mtRC activity and cartilage organization in the PFE of 1-month-old Cre and CreTW mice. Cytochrome c oxidase (CYTOCOX; complex IV)/succinate dehydrogenase (SDH) activity staining demonstrated CYTOCOX activity in articular and growth plate cartilage of 1-month-old Cre mice, with the strongest staining present at the chondro-osseus junction close to the bone marrow. In CreTW mice, loss of CYTOCOX activity was predominately observed in the calcified zone and growth plate, as indicated by blue SDH staining (Fig. 1*B*). These changes in CreTW mice were associated with the expansion of the central area of the PFE cartilage and disturbed chondrocyte organization as shown by safranin O staining (Fig. 1*C*). The results indicate that distinct chondrocyte subpopulations show a higher degree of mtRC deficiency, leading to subpopulation-specific histomorphological changes in PFE cartilage of CreTW mice.

**Figure 2:**
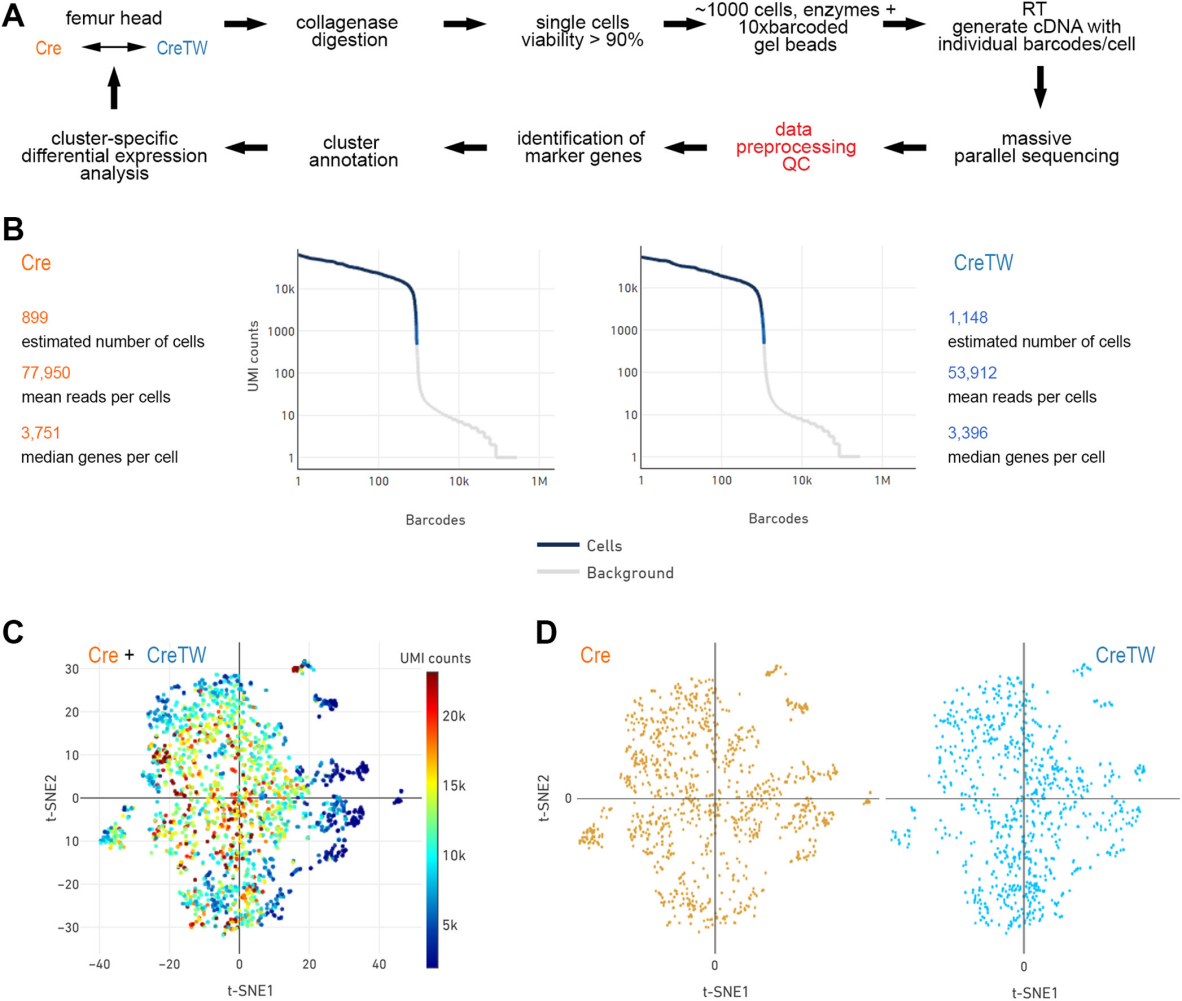
**Single-cell RNA-Seq analysis of chondrocytes isolated from PFE cartilage of Cre and CreTW mice.***A*, experimental workflow of scRNA-Seq analysis. *B*, summary of cell numbers, reads, identified genes, and count of filtered UMIs mapped to each barcode per genotype (barcode rank plots). *C* and *D*, PCA-based t-distributed stochastic neighbor embedding (t-SNE) plot of merged (*C*) and single datasets of Cre- and CreTW-derived cells (*D*). Colors indicate UMI counts (*C*) or genotype (*D*). PCA, principal component analysis; PFE, proximal femoral epiphysis; UMI, unique molecular identifier.

**Figure 3:**
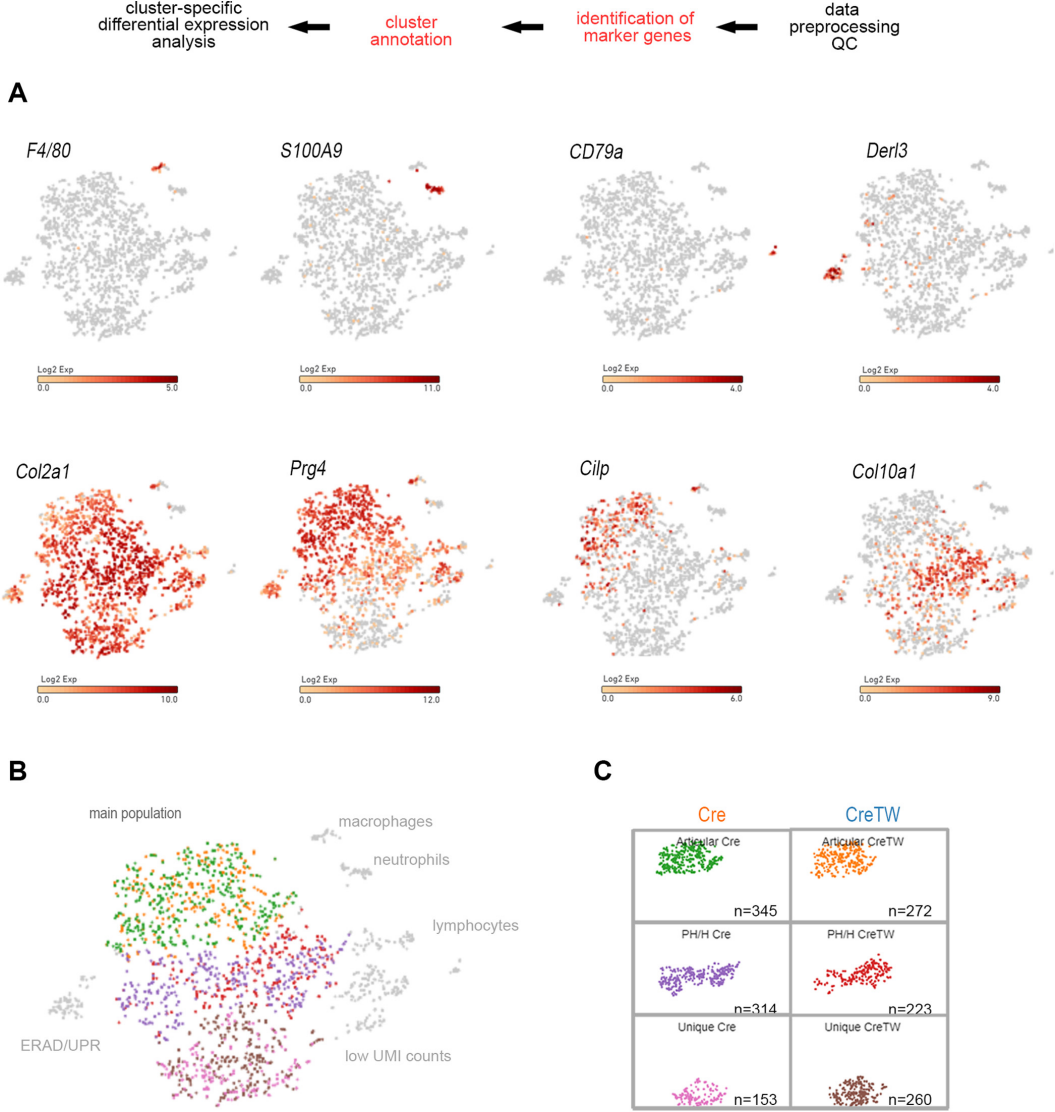
**Marker gene–based identification of cell clusters.***A*, expression of cell type–specific marker genes was used to annotate individual cell clusters. Log2 expression intensity values of individual cells are indicated (color scale). *B*, the Col2a1^+^ main population was identified, and subpopulations of articular (Cre—*green*, CreTW—*orange*), prehypertrophic/hypertrophic (Cre—*purple*, CreTW—*red*), and unique chondrocytes (Cre—*pink*, CreTW—*brown*) are highlighted. Cells with low UMI counts and noncartilaginous cells were excluded from the analysis. *C*, cluster-specific cell numbers within the *Col2a1*^*+*^ main subpopulation of Cre and CreTW mice are shown in split t-SNE plots. t-SNE, t-distributed stochastic neighbor embedding; UMI, unique molecular identifier.

scRNA-Seq analysis was then applied to correlate transcriptional changes in individual chondrocyte subpopulations of CreTW mice with the load of mtRC deficiency. Chondrocytes from PFE cartilage of 1-month-old Cre and CreTW mice were isolated by collagenase digestion, and after barcode labeling, complementary DNA (cDNA) generation, and massive parallel sequencing, the data were processed, and marker gene expression was used to annotate and analyze cluster-specific differential gene expression (Fig. 2*A*). Similar numbers of cells were analyzed, and a median of 3751 or 3396 genes per cell was identified in Cre and CreTW mice, respectively (Fig. 2*B*). High unique molecular identifier (UMI) counts (>5000 counts) were observed for the majority of cells, and several cell clusters were identified by t-distributed stochastic neighbor embedding visualization of the high dimensional transcriptome data (Fig. 2, *C* and *D*). Differential gene expression analysis was used to identify cell type–specific marker genes and to annotate individual cell clusters (Fig. 3*A*). Clusters of immune cells (*F4/80*^*+*^ macrophages, *S100A9*^*+*^ neutrophils, and *CD79a*^*+*^ B-cell clusters) and of *Derl3*^*+*^ cells linked to the endoplasmic reticulum–associated degradation pathway were detected, but the majority of cells were *Col2a1*^*+*^ chondrocytes. Within this *Col2a1*^*+*^ main population, we identified subclusters of *Prg4*^+^/*Cilp*^+^ articular, and *Col10a1*^*+*^ prehypertrophic/hypertrophic (PH/H) chondrocytes as well as a unique population of *Prg4*^−^/*Cilp*^−^/*Col10a1*^*−*^ chondrocytes (Fig. 3*B*). Interestingly, the number of cells in this unique cartilage subpopulation was increased in CreTW mice, whereas the number of cells in the articular and PH/H subpopulation was decreased (Fig. 3*C*). Therefore, mtRC deficiency may alter the cellular composition of PFE cartilage in CreTW mice.

**Figure 4:**
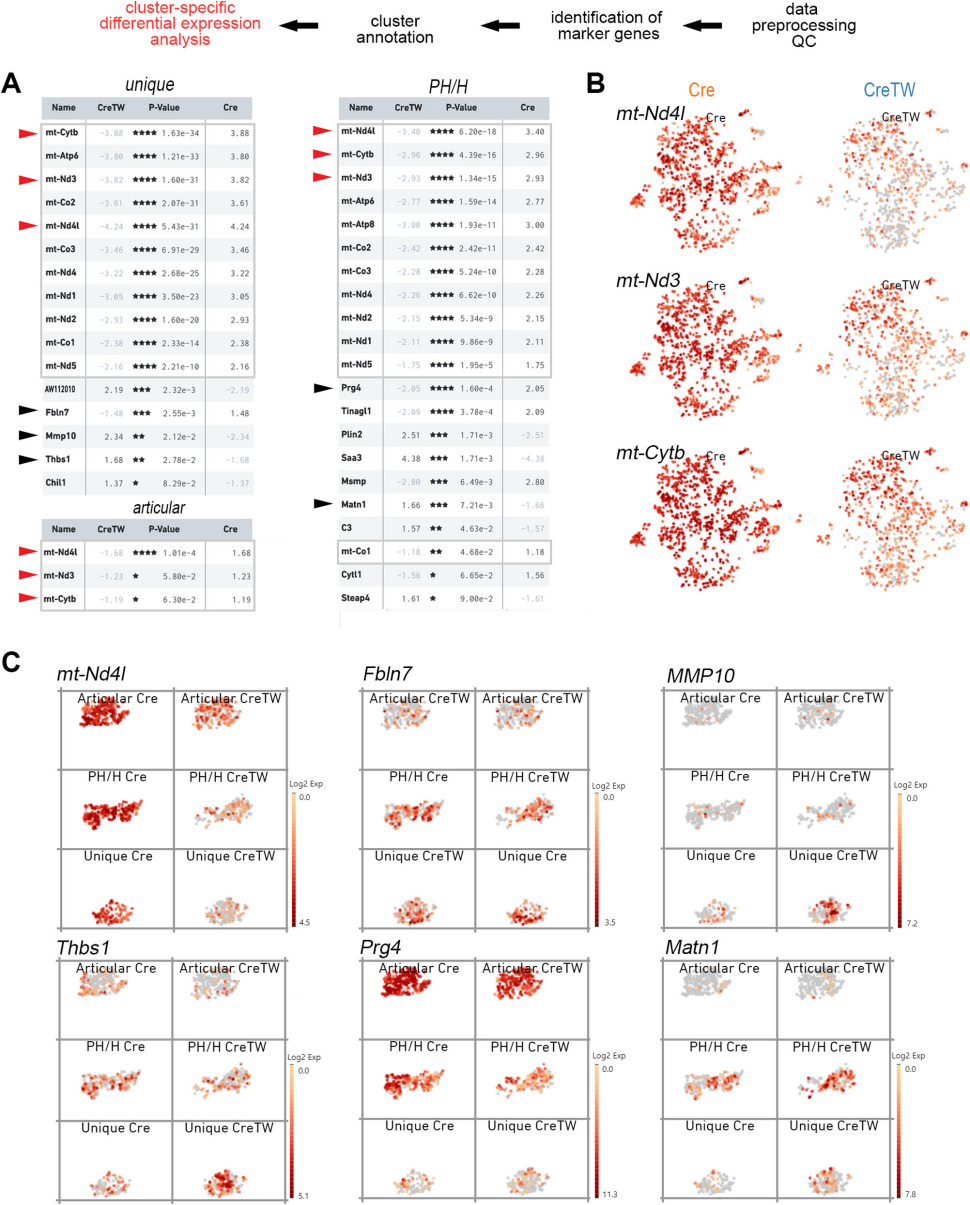
**Identification of genes regulated between chondrocyte subpopulations of Cre and CreTW mice.***A*, the transcriptome was compared between articular, prehypertrophic/hypertrophic, and unique subpopulation of PFE cartilage from 1-month-old mice using Loupe software. Regulated mtDNA-encoded genes are marked (*square*). mtDNA-encoded genes commonly regulated in all subpopulations (*red arrowhead*), and ECM-related genes are depicted (*black arrowheads*). *B*, Log2 expression intensities of regulated mtDNA-encoded genes are indicated (*color*) in t-SNE plots of single cells from Cre or CreTW mice. *C*, subpopulation-specific log2 expression values are given (*color*) in split t-SNE plots of single cells for the mtDNA-encoded mt-Nd4l gene and the nuclear-encoded ECM-related genes. ECM, extracellular matrix; mtDNA, mitochondrial DNA; PFE, proximal femoral epiphysis; t-SNE, t-distributed stochastic neighbor embedding.

Next, differential expression analysis of the chondrocyte subpopulations was performed to characterize genes that are regulated within chondrocyte subclusters of CreTW mice in response to impaired mtRC dysfunction (n = of 899 or 1.148 cells per genotype). Bioinformatics analysis uncovered 26 genes regulated between Cre and CreTW subclusters and, among those, mainly mtDNA-encoded genes were downregulated in CreTW chondrocytes (Fig. 4*A*). T-distributed stochastic neighbor embedding plots of the highest ranked differentially expressed mtDNA-encoded genes were used to visualize expression differences between chondrocyte subpopulations in Cre and CreTW mice. mt-NADH–ubiquinone oxidoreductase chain 4L (*mt-Nd4l*), mt-NADH–ubiquinone oxidoreductase chain 3 (*mt-Nd3*), and mt-cytochrome b (*mt-Cytb*) were expressed in all chondrocyte subpopulations isolated from Cre mice. Expression of these mtDNA-encoded genes was slightly reduced in articular chondrocytes of CreTW mice, and the downregulation was more pronounced in the PH/H and unique subpopulations (Fig. 4, *B* and *C*). About 14 nuclear-encoded genes were differentially expressed in nonarticular chondrocytes but not in articular chondrocytes. Five genes linked to the ECM were identified (Fig. 4, *A* and *C*), and among those, *Mmp10*, *Thbs1*, and *Matn1* were increasingly expressed in CreTW mice. The results show that mtRC dysfunction strongly affects the expression of mtDNA-encoded and nuclear ECM-related genes in nonarticular chondrocyte subpopulations.

**Figure 5:**
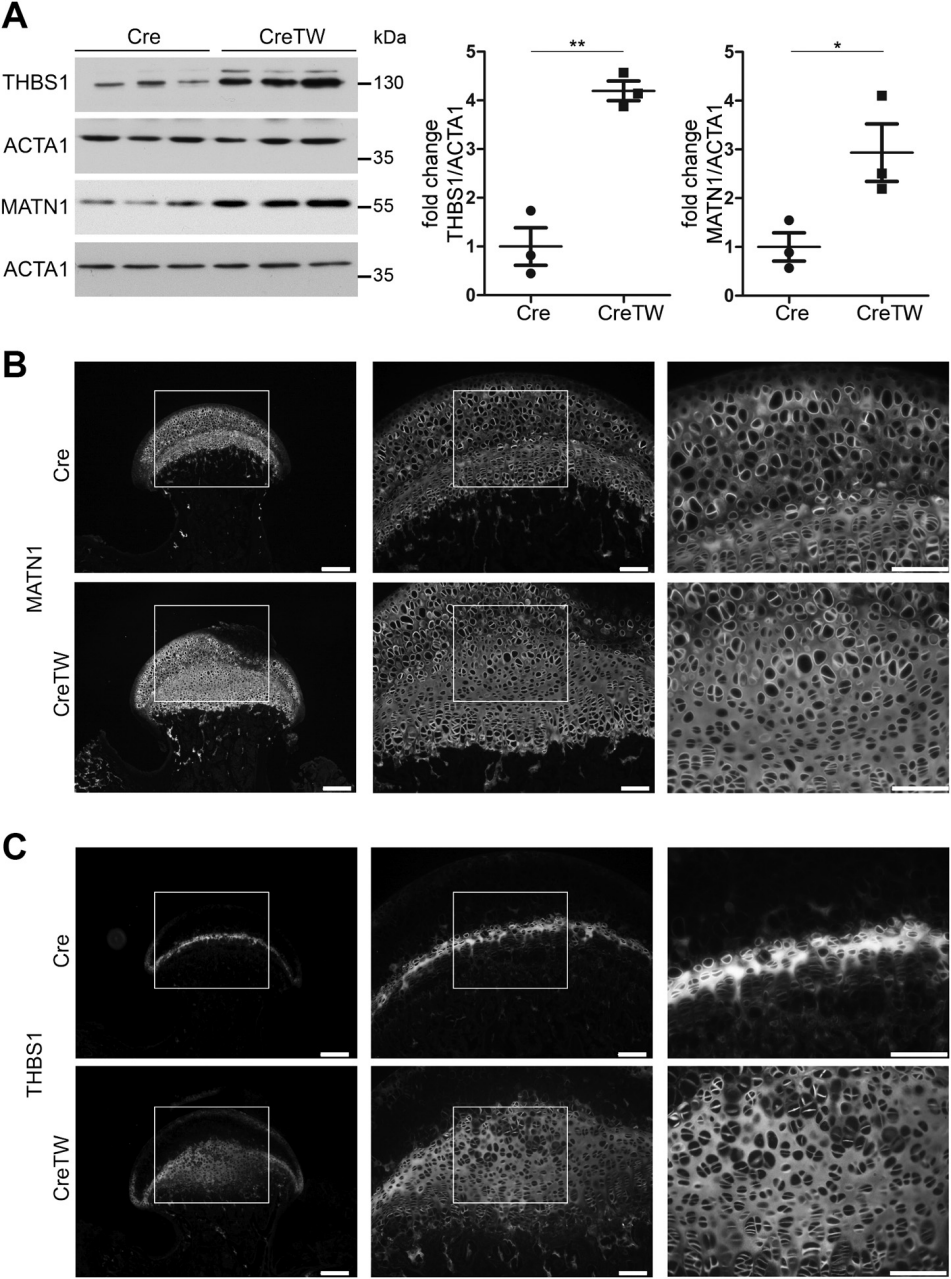
**Characterization of THBS1 and MATN1 abundance and localization in PFE cartilage of Cre and CreTW mice.***A*, THBS1 and MATN1 protein levels in cartilage extracts from 1-month-old Cre and CreTW mice were determined by immunoblotting. The fold change in protein levels in CreTW extracts compared with Cre lysates normalized to actin (ACTA1) was determined (graph). *B* and *C*, protein distribution of MATN1 (*B*) and THBS1 (*C*) in sections of PFE cartilage from 1-month-old Cre and CreTW mice was studied by immunofluorescence microscopy. Brightness was increased for visualization. The bar represents 100 μm. The brightness was adjusted for visualization. MATN1 matrilin-1; PFE, proximal femoral epiphysis; THBS1, thrombospondin 1.

To understand the consequences of this altered gene expression profile for ECM production and deposition in PFE cartilage, we studied protein abundance and distribution of matrilin-1 (MATN1) and thrombospondin 1 (THBS1) using validated antibodies. Protein levels of MATN1 and THBS1 were strongly about approximately fourfold increased in total extracts of CreTW cartilage compared with control (n = 3 per genotype), as demonstrated by immunoblot analysis (Fig. 5*A*, quantification). *In situ* detection using immunofluorescence microscopy showed that MATN1 was present in the proximal epiphysis and growth plate cartilage of Cre and CreTW mice (Fig. 5*B*) and in the expanded central area of the PFE cartilage in CreTW mice. A thin layer of extracellular THBS1 was detected at the resting zone of the growth plate cartilage in Cre mice, and this THBS1^+^ area was massively expanded into the central area of CreTW mice (Fig. 5*C*).

**Figure 6:**
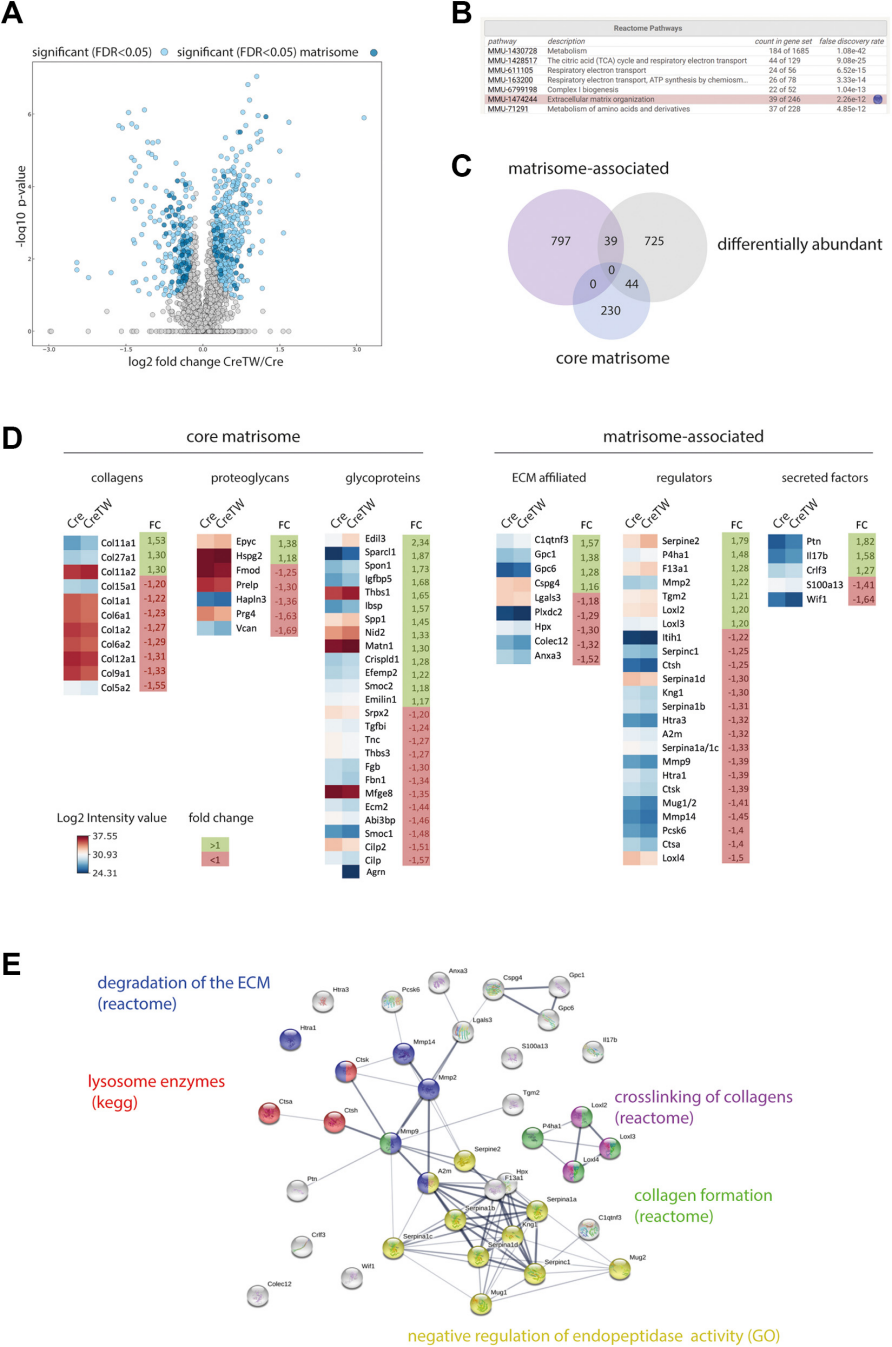
**Matrisome analysis of PFE cartilage from 1-month-old Cre and CreTW mice.***A*, volcano plot analysis of the proteome illustrating significant differences (*blue*, significant matrisome—*dark blue*) between PFE cartilage of Cre and CreTW mice (permutation-based FDR cutoff = 0.05, n = 4 biological replicates). Four individual proteomes per genotype were measured and analyzed. About 808 regulated entities were identified, including protein annotations of nonuniquely assigned peptides. *B*, reactome enrichment analysis of differentially abundant proteins. *C*, numbers of regulated core matrisome and matrisome-associated entities are determined in a Venn diagram. *D*, fold change and log2 intensity values between Cre and CreTW mice are shown. *E*, STRING analysis of matrisome-associated regulated entities using Reactome, KEGG, and GO-term pathways. FDR, false discovery rate; GO, Gene Ontology; KEGG, Kyoto Encyclopedia of Genes and Genomes; PFE, proximal femoral epiphysis.

MATN1 and THBS1 levels modulate collagen fibril assembly and ECM organization (13, 14), and a strongly increased incorporation of MATN1 and THBS1 into the ECM of CreTW mice may therefore affect the cartilage ECM organization. To determine the consequences for ECM composition, we studied the proteome of PFE cartilage from 1-month-old Cre and CreTW mice (n = 4 per genotype) using established protocols (15). In total, 808 entities were differentially abundant between PFE cartilage from Cre and CreTW mice (Fig. 6, *A* and *C*). STRING-implemented reactome enrichment analysis showed that “metabolism” and the metabolism-related terms “citric acid cycle,” “respiratory electron transport,” and “complex I” were highly enriched. “ECM organization” was the only nonmetabolism-related term within the highest ranked pathways (Fig. 6*B*), suggesting that ECM alterations are a main target of mtRC deficiency in cartilage. Therefore, we next characterized the changes in ECM composition in detail by applying matrisome analysis to the proteome dataset (16, 17). Among the regulated ECM-related entities, 44 were associated to the core matrisome and 39 to the matrisome-associated cluster (Fig. 6*C*). Collagens and proteoglycans within the core matrisome were predominately decreased in PFE cartilage of CreTW mice but not noncollagenous glycoproteins (Fig. 6*D*). Interestingly, the fibrillar collagen II and extrafibrillar proteoglycan aggrecan were detected but not regulated in proteome analysis, while we observed decreased levels of collagen II and increased levels of aggrecan by immunoblot analysis (Fig. S2*B*). In line with the previous results, the abundance of the perifibrillar protein MATN1 and the matricellular protein THBS1 was increased. In addition, 44 differentially abundant matrisome-associated proteins were identified, and STRING-based analysis showed that these proteins are primarily involved in proteolytic ECM processing, collagen formation, and crosslinking of collagen fibrils (Fig. 6*E*).

**Figure 7:**
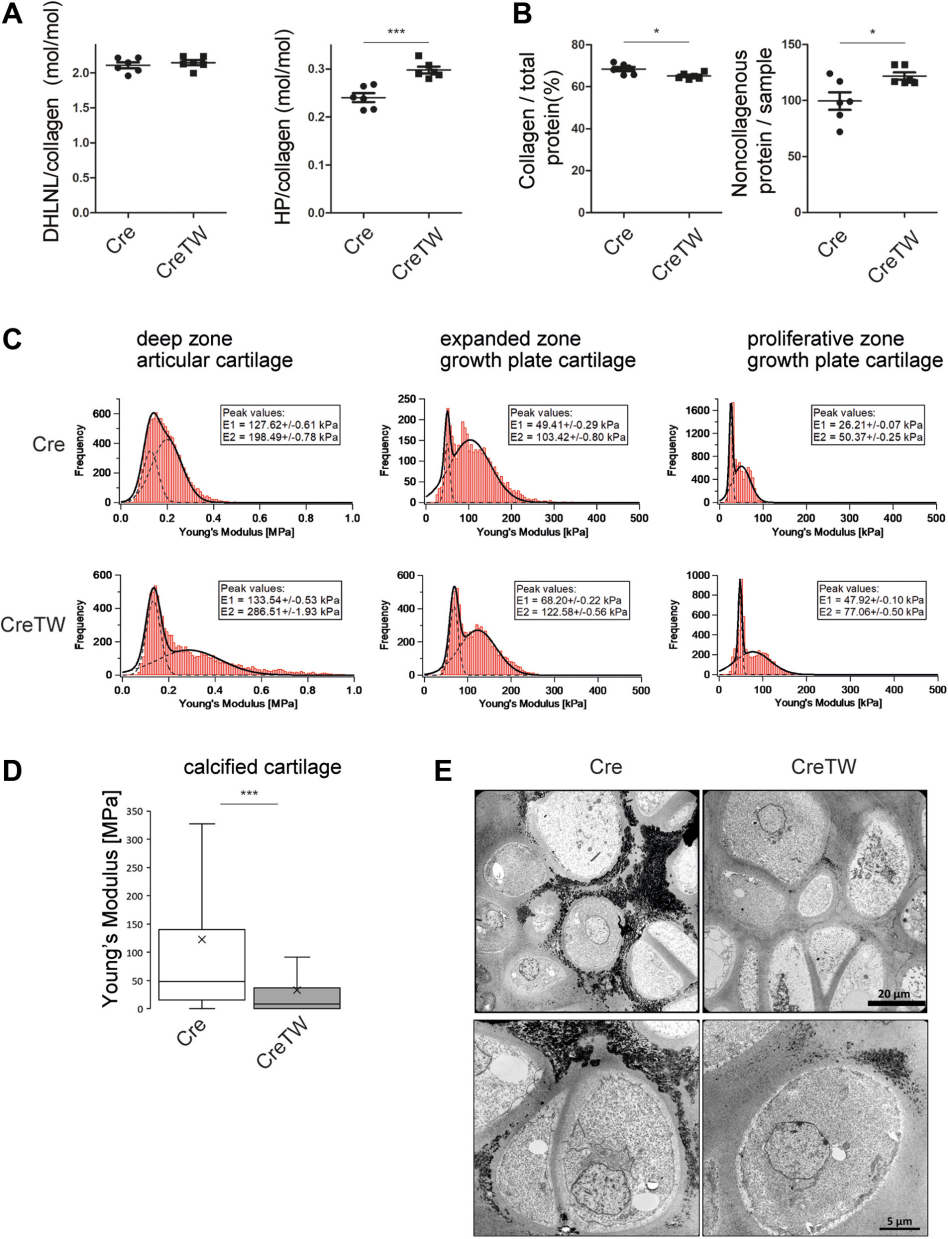
**Characterization of collagen crosslink formation and ultrastructural organization in PFE cartilage of 1-month-old Cre and CreTW mice.***A*, the difunctional crosslink dihydroxylysinonorleucine (DHLNL) and the trifunctional maturation product hydroxylysylpyridinoline (HP) in collagens and the (*B*) collagenous and noncollagenous protein content was determined by amino acid analysis (n = 6 independent biological replicates per genotype). *C*, distributions of Young's modulus values obtained by nanoindentation atomic force microscopy of native (unfixed) sections from PFE cartilage (n = 3). *Solid line*—bimodal Gaussian fit to the data (sum of two Gaussian functions); *dashed lines*—the two Gaussian functions of the bimodal fit representing the proteoglycan (first peak) and the collagen fibril moieties (second peak). The *insets* give the Young's modulus values and standard errors of the first (E1) and second peaks (E2). *D*, box plot showing median (*solid line*) and mean (*cross*), 25/75% quartile (*box*) of Young's modulus in the calcified cartilage of Cre and CreTW mice. *E*, ultrastructural analysis of the calcified cartilage. Electron dense hydroxyapatite containing matrix is detected (*black*). Overviews (*top*) and close ups (*bottom*) are shown. PFE, proximal femoral epiphysis.

Among those proteins, prolyl 4-hydroxylase and three lysyl oxidases formed a distinct protein cluster involved in collagen triple helix/fibril stability and crosslinking. Hence, we analyzed those collagen crosslinks that predominate in mature cartilage (18) and determined the content of the hydroxylysine aldehyde–derived difunctional dihydroxylysinonorleucine (DHLNL) and its trifunctional maturation product hydroxylysylpyridinoline (HP). Here, we detected a significant increase in the mature trifunctional crosslink HP in PFE cartilage of CreTW mice compared with control (n = 6 per genotype) but not of the precursor difunctional crosslink DHLNL (Fig. 7*A*). The increase in HP was associated with a slight decrease in total collagen content and an increase in noncollagenous proteins (Fig. 7*B*). The results show that crosslinking in collagen fibrils is increased in mtRC deficient cartilage, which may affect the stiffness and mechanostability of the ECM in cartilage.

Changes in compressive stiffness of the ECM (Young's modulus) were then studied by nanoscale indentation-type AFM at various regions of the PFE (n = 3 per genotype). Bimodal distributions of the Young’s modulus were observed in the deeper articular zone, expanded zone, and proliferative zone of the growth plate. The two peaks in the distributions represent the softer proteoglycans (E1) and the stiffer collagens (E2) (Fig. 7*C*). The ECM of 1-month-old PFE cartilage from CreTW mice showed a general 20 to 50% increase in stiffness of the collagen moiety (E2) in all three regions investigated, whereas the proteoglycan moiety (E1) was stiffer only in the nonarticular resting and proliferative zone of growth plate cartilage. Unimodal distribution was detected in the much stiffer calcified cartilage zone, and the Young's modulus distributions exhibited only one distinct peak for Cre and CreTW, respectively (histograms not shown). Here, the median Young's modulus in CreTW mice was decreased by more than 80% in CreTW mice compared with control (Fig. 7*D*). ECM stiffness and mineralization processes are closely linked (19, 20), and we hypothesized that the decreased ECM stiffness in CreTW mice may indicate a reduced incorporation of hydroxyapatite crystals into collagen fibrils and less biomineralization. We therefore used electron microscopy to study the mineralization in the calcified zone of PFE cartilage. Electron dense mineralized structures were found in the ECM of chondrocytes in both Cre and CreTW mice (Fig. 7*E*). However, in Cre mice, the chondrocytes were surrounded by black needle-like crystal structures, and these mineralized areas were markedly reduced in CreTW mice.

In summary, mtRC dysfunction in PFE cartilage induces a subpopulation-specific ECM-damage response in growth plate cartilage that alters the balance between collagenous and noncollagenous proteins, increases ECM crosslinking and stiffness, and reduces mineralization in the calcified zone.

## Discussion

The mtRC is a surprisingly major prerequisite for postnatal cartilage growth and differentiation, but the requirement of mtRC for ECM homeostasis in mature cartilage has not yet been defined. Here, we show by scRNA-Seq analysis that impaired mtRC leads to a cell subpopulation–specific ECM-damage response and an altered mechanostability in PFE cartilage.

### Marker gene annotation can identify chondrocyte subclusters in mature PFE cartilage

The visualization of the transcriptome landscape at the single-cell level in a heterogeneous musculoskeletal cell population is technically challenging but was previously achieved for the developing knee joint and growth plate cartilage (8, 9, 10). Here, we successfully applied scRNA-Seq analysis to mature cartilage from 1-month-old TW helicase K320E transgenic mice. Similar cell numbers were sequenced, and similar cell types were identified in the PFE cartilage of Cre or CreTW mice, indicating that the isolation procedure has no influence on the sample composition. Because of the close association of bone marrow and cartilage at the chondro-osseus junction, a minor proportion of immune cells attached to the dissected PFE cartilage was found. Interestingly, F4/80^+^ macrophages were present within the immune cell population that expresses cartilage-specific ECM transcripts, for example, *Prg4*, *Cilp*, and *Col2a1*. This population may correspond to the nondestructive synthetic macrophages previously described to produce almost all known collagen mRNAs and to be involved in tissue remodeling and repair (21). Within the *Col2a1*^+^ main chondrocyte population, articular and PH/H subpopulations could be identified by marker gene expression analysis. *Prg4* (22) and *Cilp* (23) are common markers of articular cartilage, and both transcripts were expressed in PFE cartilage, but *Cilp* expression is restricted to a subpopulation of *Prg4*^+^ cells. This reflects the *in situ* expression of *Cilp* and *Prg4* in cartilage, where *Cilp* is detected in the intermediate zone of articular cartilage (24), whereas *Prg4* is expressed in the intermediate and superficial zone of articular cartilage (22, 25). *Col10a1* mRNA is expressed by PH/H chondrocytes (12) and was used to define the PH/H subpopulation within the scRNA-Seq dataset. Finally, a unique population of nonarticular *Thbs1*^*+*^chondrocytes was detected and linked to the expanded and disorganized central region of PFE in CreTW mice *in situ*. THBS1 was previously detected in a particular subpopulation of chondrocytes at a thin zone directly adjacent to mature PFE growth plate (26), presumably cells of the resting zone, and this subpopulation was also found by scRNA-Seq analysis. The results suggest that the resting zone is expanded in response to mtRC dysfunction with multiple clusters of cells and no evident columnar organization in PFE cartilage. These findings differ from our previous results where mainly the hypertrophic area in the distal femoral epiphysis was expanded (6). However, the proximal and distal epiphyses show significant differences in development. In mice, unlike most mammalian species (including human), the proximal epiphysis in the hip joint develops with no secondary ossification center forming in the epiphysis, but with direct mineralization of the cartilage (27). In contrast, a secondary ossification center develops in the distal femoral epiphysis of the knee joint, and vessels can provide oxygen and nutrients to both ends of the growth plate. Therefore, cells in the resting zone of the PFE in CreTW animals may show a unique damage response to mtRC dysfunction compared with the knee joint epiphysis. Interestingly, scRNA-Seq analysis is suited to identify these cells and characterize their unique transcriptional response, and we found an increase in cell numbers of the unique populations in CreTW mice. This indicates that scRNA-Seq analysis also provides quantitative information on the cellular composition of tissue samples similar to flow cytometry experiments. scRNA-Seq analysis may therefore provide as a combined tool to determine individual transcriptomes and quantify cell populations to study regulated programs and changes in population composition.

### scRNA-Seq analysis quantifies mtRC deficiency in chondrocyte subclusters

We observed a downregulation of mt-DNA–encoded subunits of mtRC complexes in CreTW mice compared with control. Interestingly, the decrease in expression was more pronounced in PH/H and unique chondrocyte subpopulations, which points to differences in mtDNA deletion/depletion-mediated impairment of mt-DNA encoded gene expression between chondrocyte subpopulations in CreTW mice. TW K320E expression is driven by the Col2a1 promoter (4), and higher UMI counts of the collagen II transcripts were detected in nonarticular cartilage. Therefore, Col2a1-driven TW K320E expression levels could be higher in nonarticular cartilage, leading to a more pronounced mt-DNA depletion/deletion and impaired mtRC complex gene expression compared with articular cartilage. In addition, TW mutant–mediated mtDNA depletion/deletion requires cell division (2), but articular chondrocytes rather grow by cell volume expansion and topographical cell rearrangement (25). As a consequence, mtDNA deletion/depletion could be more efficient in nonarticular subpopulations. This is supported by our observation that mtRC inhibition was more pronounced in nonarticular cartilage compared with articular cartilage, and only nonarticular subpopulations show significant differences in nuclear-encoded gene expression.

The limitations of this study include that despite the severe damage in PFE cartilage of CreTW mice, only few non–mtDNA-encoded genes were identified. This is a potential drawback of the scRNA-Seq analysis compared with bulk transcriptome approaches. Both methods require amplification when using small amounts of mRNA, but cell lysis, reverse transcription, and amplification of RNA in a single cell substantially increases technical noise relative to bulk-level RNA-Seq (28). The detection of lowly expressed genes can also be hindered, and low data quality may be obtained from cells damaged or dying after isolation. We could observe a median of ∼3500 expressed genes per cell by scRNA-Seq of PFE cartilage. While scRNA-Seq analysis has a lower sensitivity of transcript detection compared with RNA bulk sequencing, scRNA-Seq provides unique information on subcluster-specific expression changes. Our study shows that analysis of mt-encoded gene expression correlates well with the load of mtRC damage in chondrocyte subpopulations and with *in situ* histomorphological changes in PFE cartilage. Hence, scRNA-Seq analysis can monitor the onset and progression of mtRC deficiency in cartilage and presumably also in other tissues.

### mtRC deficiency alters ECM composition and increases matrix stiffness

Using scRNA-Seq analysis, we identified the ECM as a central component of the transcriptional response to mtRC deficiency in nonarticular cartilage of CreTW mice. Here, scRNA-Seq analysis provided a high cellular resolution to define individual cellular responses, but a combined experimental validation approach by immunoblot, immunofluorescence microscopy, and mass spectrometry/matrisome analysis was essential to validate and expand the results of the scRNA-Seq analysis. Specifically, mass spectrometry analysis provides important information on matrisome-associated proteins that are primarily involved in proteolytic ECM processing, collagen formation, and crosslinking of collagen fibrils but could not be detected by scRNA-Seq analysis. We therefore recommend this combination of methods to provide a comprehensive view on ECM changes in response to mtRC dysfunction.

*Mmp10* and *Thbs1* are both upregulated in scRNA-Seq and mass spectrometry analysis experiments and may contribute to the mitochondrial damage response. MMP10 is known to be induced upon injury (29) and presumably upon mitochondrial dysfunction (30). This proteinase is found at sites of resorption in the growth plate (31) and activates procollagenases relevant for ECM remodeling (32) and tissue repair processes (33). Therefore, MMP10 may accumulate in the cartilage area with a strong impairment of mitochondrial function to activate collagenases and drive the expansion of the central area of the femoral head cartilage. In addition, the increased production of the matricellular protein THBS1 and the perifibrillar adaptor protein MATN1 can affect ECM formation and homeostasis. THBS1 not only directly interacts with collagen fibrils and lysyl oxidase precursors to regulate the organization of collagen fibril packing (14) but also inhibits the activation of metalloproteinases (34). Increased THBS1 levels are associated with aging during which the ECM becomes stiffer, whereas mice lacking THBS1 show reduced collagen crosslinking in skin (14). We observed increased expression and deposition of THBS1 in the ECM of nonarticular cartilage of CreTW mice, which was associated with an increase in collagen crosslinks and ECM stiffness. Presumably, THBS1 directly increases the ECM stiffness of CreTW mice by modulating collagen packing and crosslinking. We also detected a slight decrease in total collagen content and an increase in noncollagenous proteins, which is in line with our finding that the noncollagenous proteins THBS1 and MATN1 are increased in response to mtRC damage. MATN1 interconnects and stabilizes the fibrillar and extrafibrillar components of the ECM, and its genetic deletion in mice leads to disturbed type II collagen fibrillogenesis and fibril organization (35). Thus, increased MATN1 deposition in the cartilage ECM of CreTW mice may also induce changes in fibril organization and increased ECM stiffness. mtRC deficiency induces additional changes in the matrisome organization and altered abundance of proteins involved in proteolytic ECM processing, collagen formation and crosslinkings are most evident, as shown by STRING-based bioinformatics analysis. A complex interplay between induced THBS1, MATN1, and matrisome-associated components involved in cartilage ECM formation and remodeling may shift the balance to a stiffer cartilage ECM in CreTW mice.

Because of their direct involvement in ECM organization, THBS1 and MATN1 may deliver mechanical and biochemical cues of the ECM to influence cell and tissue-specific responses to mtRC deficiency in cartilage. THBS1 expression is induced in response to injury and interacts with integrins to sense and deliver mechanical cues to the cell (36, 37). The matricellular THBS1 was shown to prevent hypertrophic chondrocyte differentiation and bone formation in cartilage repair experiments (38). Its upregulation and increased deposition in the ECM of CreTW mice may provide signals to prevent premature hypertrophic differentiation and ossification of PFE cartilage in mtRC deficiency. THBS1 deposition is associated with the expansion of the resting chondrocytes in central region of PFE in CreTW mice and the increase in THBS1, and some of the observed ECM pertubations could also be a result of changes in the proportion of chondrocyte populations rather than consequences of altered ECM production, secretion, or remodeling. Hence, population imbalances may also contribute to ECM pertubations in response to mtRC dysfunction in PFE cartilage. ECM changes may be sensed by resting chondrocytes to affect cell proliferation and survival, and we examined the number of proliferating cell nuclear antigen (PCNA^+^) proliferating cells and TUNEL^+^ dying cells in PFE cartilage of 1-month-old Cre and CreTW mice. Here, we could observe increased numbers of late hypertrophic chondrocytes at the chondro-osseus border that struggle to survive (Fig. S2). These findings are in line with our results that cell death is induced at the cartilage–bone junction of the distal femoral epiphysis, but changes in proliferation or cell death were apparently not detected in the THBS1^+^ area of PFE cartilage. Therefore, increased abundance of THBS1 does not cause any changes in cell proliferation or survival of resting chondrocytes. In addition, THBS1 and MATN1 are described to be potent endogenous antiangiogenic factors suppressing capillary endothelial cell proliferation and migration (39, 40), and, interestingly, decreased levels of THBS1 and MATN1 are associated with premature vascularization and ossification in PFE cartilage of collagen IX-deficient mice (26). Both proteins could exert an antiangiogenic function to inhibit premature vascularization and ossification of PFE cartilage in CreTW mice, which may also explain the decreased chondrocyte-mediated mineralization of the ECM observed by ultrastructural analysis. This is in line with that we could not detect any vessel infiltration (Fig. 1) or increase in vascular endothelial growth factor (VEGF) mRNA expression, protein abundance, and secretion in chondrocytes from 1-month-old PFE cartilage of CreTW mice (Fig. S1, *C*–*E*). Thrombospondins and matrilins may have physiological roles in the regulation of inflammatory reactions (41, 42), and we could not observe an inflammatory response in chondrocytes with mtRC dysfunction (Fig. S1, *A* and *B*). These findings may support the view that THBS1 and MATN1 suppress an inflammatory response in response to mtRC dysfunction in cartilage, but further molecular studies are needed.

The effects of metabolism on ECM organization are poorly understood (1), and we now use a combined approach of high-resolution scRNA-Seq, mass spectrometry, and matrisome analysis to show that ECM homeostasis in mature growth plate cartilage requires mtRC. We demonstrate that mtRC failure induces an ECM-specific damage response leading to increased collagen crosslinking and matrix stiffness. Hence, scRNA-Seq identified mtRC deficiency as a major cue for impaired ECM integrity and mechanostability in cartilage *in vivo*.

## Experimental procedures

### Animals

Col2a1-Cre C57BL/6N mice were crossed with R26-K320E Twinkle^loxP/+^ mice to generate heterozygous Col2a1-Cre-26-K320E-Twinkle^loxP/+^ C57BL/6J mice (CreTW) expressing Tw^K320E^ mutant helicase in cartilage and Col2a1-Cre C57BL/6 (Cre) control mice (5). All experiments were performed with female mice in agreement with the guidelines of the German animal protection law (Institutional Review Board: ”Landesamt für Natur, Umwelt und Verbraucherschutz Nordrhein-Westfalen”). Animals were housed in a pathogen-free facility at 20 to 24 °C on a 12 to 12 h light/dark cycle in individual ventilated cages and supplied with standard irradiated mouse chow and water *ad libitum*.

### Histology and CYTOCOX/SDH staining

Femora were isolated and fixed with 4% paraformaldehyde for 18 h. Fixed samples were decalcified using 0.5 M EDTA (pH 8.0), embedded in paraffin, and sectioned into 7-μm sections using a microtome (HM355 S; Thermo Fisher Scientific). The morphological organization of the PFE was evaluated on deparaffinized sections by safranin O staining (0.1% safranin O; Sigma–Aldrich). Activity of the mitochondrial complex IV (CYTOCOX) and complex II (SDH) was assessed on cryoembedded tissue. Isolated cartilage of the proximal femoral end was embedded in optimal cutting temperature compound medium (Tissue-Tek; Sakura), shock frozen in liquid nitrogen, and sectioned using the CM3050 cryostat (Leica Biosystems). About 7-μm cryosections were stained with a 1 mg/ml 3,3-diaminobenzidine (Sigma–Aldrich) solution for 30 min at 37 °C to visualize CYTOCOX activity (43). After washing with PBS, sections were treated with 2 mg/ml nitrotetrazolium blue chloride (Sigma–Aldrich) solution containing 0.2 M sodium succinate (Sigma–Aldrich) and 50 mM MgCl_2_ (Merck KGaA) for 2 h at 37 °C to detect SDH activity. Stained sections were embedded in Kaiser's glycerol gelatine (Merck KGaA) and analyzed using a Nikon Eclipse TE2000-U microscope (Nikon).

### Single-cell isolation and RNA-Seq

Isolated PFE cartilage was chopped and incubated at 37 °C under constant rotation (HBMSUV-14 Hybaid Hybridization Oven Maxi 14) using collagenase P solution (1.61 U/ml collagenase P; Roche Diagnostics GmbH) in Dulbecco's modified Eagle's medium F12 (Life Technologies Europe B.V.) containing 1% penicillin/streptomycin (Life Technologies Europe B.V.), 10% fetal calf serum (Biochrome GmbH), and 0.1755 mg/ml cysteine (Sigma–Aldrich). After 18 h, the cell suspension was transferred through a 40-μm cell strainer and washed with 1.5% bovine serum albumin (Serva Electrophoresis GmbH) in PBS (Life Technologies Limited). Cells were centrifuged at 400*g* for 5 min (Eppendorf Centrifuge 5415R), resuspended in 200 μl 1.5% bovine serum albumin/PBS (200 cells/μl), and cell viability (>90%) was confirmed by trypan blue staining. cDNA was prepared according to the 10× Genomics Chromium Single Cell 3′ Reagent Kit User Guide (v3Chemistry) from the pool of ∼1000 cells. At this point, all cDNA fragments carried the 16 nt cellular barcode and the 12 nt long UMIs at the poly(dT) end. About 25% (10 μl) of this cDNA solution continued with the original 10× Genomics protocol to create an Illumina 3′mRNA library with P5 and P7 Illumina adapters. Sequencing was done on Illumina NovaSeq6000 systems with a two-step approach (to validate cell numbers, UMI count, and saturation) finally leading to an estimated number of cells of 899 or 1148, 77,950 or 53,912 mean reads per cell, and 3751 or 3396 medium genes per cell, respectively. Cell ranger analysis (10× genomics) was done using the 3.1.0 pipeline version for Loupe Browser 4.1.0 evaluation of the data.

### Quantitative real-time PCR and semiquantitative PCR

RNA from PFE cartilage of 1-month-old mice was reversely transcribed into cDNA using the Omniscript RT assay (Qiagen). For semiquantitative PCR, the REDTaq ReadyMix (Sigma–Aldrich) and the *Vegfa* primer (forward: 5′-CTGCTCTCTTGGGTGC ACTG-3′; reverse: 5′-C ACCGCCTTGGCTTGT CACA-3′) were used. mRNA expression levels were determined by quantitative real-time PCR using the SYBR Green assays (Thermo Fisher Scientific). For quantitative real-time PCR, the fold change was calculated with the ΔΔCT method. The following primers were used: *Actb* (forward: 5′-GACGAGGCCCAGAGCAAGAG-3′; reverse: 5′-CTAGAGCAACATAGCACAGC-3′), *Il-1beta* (forward: 5′-TTGACGGACCCCAAAA GAT-3′; reverse: 5′-GATGTGCTGCTGCGA GATT-3′), and *Vegfa* (forward: 5′-GCAGCTTGAGTT AAACGAACG-3′; reverse: 5′-GGTTCCCG AAACCCTGAG-3′).

### Mass spectrometry analysis

PFE cartilage of three animals was lyophilized and then processed to powder using 2.8-mm ceramic beads (CK28 Hard tissue homogenizing tubes, Precellys tissue homogenizer; Bertin) at room temperature (RT) for 3× 20 s at 6800 rpm with 30 s pause in between. The powder was subsequently lysed in 200 μl precooled lysis buffer (4% [w/v] SDC and 100 mM Tris–HCl [pH 8.5]) and then incubated at 95 °C for 5 min and sonicated using a tip-probe sonicator (SONOPULS HD2070; Bandelin) for three 30-s cycles of 1 s on and 1 s off at 80% output. Debris was removed by centrifugation at 16,100*g* (Eppendorf 5430 R-Centrifuge) for 10 min at RT, and protein concentration was determined by Pierce 660 nm Protein Assay (Thermo Fisher Scientific). Lysates were reduced and alkylated with 100 mM Tris(2-carboxyethyl)phosphine (Pierce, Thermo Scientific) and 400 mM 2-chloroacetamid (pH 7–8) (Sigma–Aldrich; Merck) for 5 min at 45 °C under constant movement (1500 rpm; Eppendorf ThermoMixer C). After enzymatic digestion with Lys-C (1 mg/ml) (Wako) and trypsin (1 mg/ml) (Sigma–Aldrich; Merck) using an enzyme-to-substrate ratio of 1:100 (w/w) at 37 °C with constant shaking (1500 rpm; Eppendorf ThermoMixer C), 10 μg of the sample were transferred with 100 μl loading buffer (1% TFA in isopropanol) onto SDB-RPS stage tips (AttractSPE Disks SDB-RPS; Affinisep). Stage tips were washed, and peptides were eluted with 0.25% NH_4_OH in 60% acetonitrile. The eluate was concentrated under vacuum to dryness using a speed vac concentrator (Concentrator Plus; Eppendorf), reconstituted with 10 μl resuspension buffer (5% formic acid and 2% acetonitrile) and measured by LC–MS/MS (Q Exactive Plus; Thermo Fisher Scientific) as described previously (44). Raw files were processed using MaxQuant (version 1.5.3.8) and its implemented Andromeda search engine and LFQ algorithm. The mouse UniProt-FASTA database (June 2017 with 16,890 entries) was used for protein and peptide identification. Specific digestion with trypsin was selected, and maximum two missed cleavages were permitted. Carbamidomethylated cysteine was used as a fixed modification and oxidation (M), acetylation (N-terminal), and phosphorylation of a serine, threonine, or tyrosine were included as variable modifications. Mass tolerance for precursor ions was 20 ppm for first and 4.5 ppm for the main search and 20 ppm for fragment ions. Spectra were also searched against a reverse decoy database, and a false discovery rate (FDR) of 1% was applied on peptide and protein level. Perseus (version 1.5.5.3; Max Planck Institute of Biochemistry) was used to perform the two-sided Student's *t* test and calculate permutation-based FDR (S_0_ = 0.1; number of randomizations = 500). The volcano plot was generated using Instant Clue software 0.5.2 (University of Cologne) (45).

### Immunofluorescence and immunohistochemistry analysis

In-plane matched paraffin sections (7 μm) of PFE cartilage were incubated with 5 mg/ml hyaluronidase (Sigma–Aldrich) in hyaluronidase buffer (0.1 M NaH_2_PO_4_, 0.1 M NaAc, and pH 5.0) for 30 min at 37 °C. Sections were washed with Tris-buffered saline and digested with 10 μg/ml proteinase K (Sigma–Aldrich) using proteinase K buffer (50 mM Tris–HCl, 1 mM EDTA, and pH 7.4) for 10 min at 50 °C. Mouse anti-THBS1 (1:100; 20 μg/ml, sc-59887; Santa Cruz Biotechnology) or rabbit anti-MATN1 (1:500; (46)) or rabbit anti-S100A9 (1:500; PA1-46489; Thermo Fisher Scientific) were first incubated at 4 °C overnight and then with corresponding Cy3-coupled secondary antibodies (1:800; Jackson ImmunoResearch) and 4′,6-diamidino-2-phenylindole (Sigma–Aldrich) for 1 h at RT. Sections were embedded in Mowiol and analyzed in a blinded manner using Nikon Eclipse TE2000-U microscope (Nikon). To assess the distribution of PCNA, immunohistological stainings were performed. In-plane matched paraffin sections (7 μm) of PFE cartilage were pretreated with citrate buffer (pH 6) for 1 h at 60 °C. Sections were washed with 0.1% Triton, and endogenous peroxidase activity was blocked using 3% H_2_O_2_ for 10 min at RT. After washing with 0.1% Triton, sections were incubated with a rabbit anti-PCNA primary antibody (1:200, ab2426; Abcam) overnight. An appropriate secondary antibody labeled with horseradish peroxidase (Zytomed Systems) was applied for 1 h at RT, and AEC Solution (Zytomed Systems) was used for detection. PCNA-positive chondrocytes per square millimeter were determined using ImageJ software (version 1.53; National Institutes of Health and the Laboratory for Optical and Computational Instrumentation).

### Fluorometric TUNEL assay

To evaluate the number of apoptotic chondrocytes on paraffin sections (7 μm) of PFE cartilage, the DeadEnd Fluorometric TUNEL Assay (Promega) was performed according to the manufacturer's instructions. Sections were digested with 20 μg/ml proteinase K (Sigma–Aldrich) for 8 min at RT, fixed with 4% paraformaldehyde for 10 min, and incubated in equilibration buffer for 10 min at RT. Sections were incubated for 1 h at 37 °C with the rTdT labeling reagent mix and then washed with 2xSSC buffer and PBS. 4′,6-Diamidino-2-phenylindole was used to visualize nuclei. TUNEL-positive chondrocytes and the total cell population were quantified using ImageJ (version 1.53).

### Immunoblot analysis

Isolated PFE cartilage of three individual animals per genotype or cultured cells was taken up in lysis buffer (20 mM Tris–HCl, pH7.4, 150 mM NaCl, 1% NP-40, 0.05% Triton X-100, and 0.5% sodium deoxycholate) containing cOmplete Protease Inhibitor (Roche Diagnostics GmbH), and sonification was carried out at RT with 20-s cycles and 50% output (Branson Sonifier 450; Thermo Fisher Scientific). PFE cartilage extracts were incubated overnight at 4 °C. About 20 μl of the lysates were separated by 10% SDS-PAGE, proteins were transferred to a nitrocellulose membrane (Amersham Protran 0.45 μm NC, GE Healthcare Life Sciences). For slot blot analysis, chondrocytes were isolated from PFE cartilage extracts and cultured for 3 days in Dulbecco's modified Eagle's medium F12 containing 10% (v/v) fetal calf serum, 1% (v/v) penicillin/streptomycin. About 500 μl of the supernatant was collected, loaded onto the slots, as well as the VEGF-A standard (R&D Systems), and transferred to a nitrocellulose membrane using vacuum. The nitrocellulose membrane was incubated with 5% milk powder in TBS containing 0.1% Tween-20 for 1 h at RT. Primary antibody against THBS1 (1:1000; 20 μg/ml, sc-59887; Santa Cruz Biotechnology), MATN1 (1:500; (46)), ACAN (1:500, AB1031; Merck Millipore), COL2 (1:500, CP18; Merck Millipore), VEGF-A (1:1000, sc-7269; Santa Cruz Biotechnology), and a-ACTA1 (1:200; 1 mg/ml; Merck Millipore) and appropriate horseradish peroxidase–labeled secondary antibodies (DAKO) were applied. Chemoluminescence reaction and X-ray films (Agfa Healthcare) were used to visualize specific binding. Relative protein levels were determined in a blinded manner using ImageJ software (version 1.53).

### Crosslink analysis

To determine collagen amount and crosslinks, PFE cartilage was reduced by sodium borohydride (Sigma; 25 mg NaBH_4_/ml in 0.05 M NaH_2_PO_4_/0.15 M NaCl, pH 7.4, 1 h on ice, 1.5 h at room temperature) to stabilize acid-labile collagen crosslinks. Thereafter, the samples were hydrolyzed in 6 N HCl at 110 °C for 24 h. The hydrolysates were precleared by solid phase extraction to remove the bulk of noncrosslinked amino acids (Aspec). Dried eluates were redissolved in sodium citrate loading buffer (pH 2.2) and analyzed on an amino acid analyzer (Biochrom 30; Biochrom) using a three buffer gradient system and postcolumn ninhydrin derivatization. The column was eluted for 5 min (flow rate of 15 ml/h) with sodium citrate buffer (pH 4.25), for 40 min with sodium citrate buffer (pH 5.35), and for 20 min with sodium citrate/borate buffer (pH 8.6) at 80 °C. Retention times of individual crosslinks were established with authentic crosslink compounds. Quantitation was based on ninhydrin-generated leucine equivalence factors (DHLNL 1.8; HP: 1.7) (47). Collagenous and noncollagenous protein contents of the samples were analyzed in an aliquot of hydrolyzed specimens prior to solid phase preclearance by amino acid analysis. Collagen content was calculated based on a content of 14 mg hydroxyproline in 100 mg collagen. The nomenclature of the collagen crosslinks used in the article refers to the reduced variants of crosslinks (DHLNL).

### AFM

PFE cartilage was embedded in optimal cutting temperature compound medium (Tissue-Tek; Sakura) and snap frozen using liquid nitrogen–cooled isopentane. A Cryotome (CM1850; Leica Biosystems) was used to prepare 20-μm sections covered by adhesive tape (Tesa Film no.: 57330-00000). Immediately after cutting, the sections were fixed on 2 cm × 6 cm microscope slides (Thermo Fisher Scientific) using double-sided adhesive tape (Tesa Film no.: 56661-00002) inside the cryotome as described (48) and then stored at −20 °C. Indentation-type AFM was performed as previously described (49) in a blinded manner. Briefly, the 20-μm sections were thawed, immersed in PBS (Biochrom Dulbecco's PBS without Mg^2+^/Ca^2+^, pH 7.4), and transferred to the AFM (NanoWizard; JPK Instruments), which was combined with an inverted optical microscope (Axiovert 200; Carl Zeiss) to identify the area of interest for indentation-type AFM measurements. The calcified area was identified by phase contrast microscopy to position the nanoindentation needle in the area of interest. The setup was mounted on an active vibration isolation table (Micro 60; Halcyonics) inside a 1 m³ soundproof box. Indentation experiments were carried out using silicone-nitride cantilevers (MLCT, Cantilever E, Bruker) with a nominal spring constant of 0.1 N/m, a nominal tip radius of 20 nm, and a pyramidal tip shape. For each cantilever, the spring constant was determined individually prior the AFM measurements using the thermal noise method. For each area of interest, three grids of 3 × 3 μm were assessed. Within each grid, at 25 × 25 equally spaced positions, force–indentation curves were recorded. The indentation depth was limited to 500 nm. The Hertz–Sneddon model was used as implemented in the JPK Data Processing Software (version 5.0.96; JPK Instruments) to extract the Young's modulus from each indentation curve. Next, Young's modulus distributions were generated and visualized as histograms using Igor Pro software (version 6.3.7.2; WaveMetrics). Finally, a bimodal Gaussian distribution (sum of two Gaussian functions) was fitted to each dataset (50), using a least squares fit algorithm, and displayed together in the histogram using Igor Pro software (Table S1).

### Electron microscopy

To evaluate the ultrastructure of the PFE, transmission electron microscopy was utilized. Isolated PFE cartilage was incubated with 2.5% glutaraldehyde (Serva) in 0.1 M phosphate buffer (pH 7.4) for 24 h at 4 °C, washed briefly in 0.1 M Sörensens phosphate buffer, and treated with 2% osmium tetroxide/PBS (Sigma–Aldrich) for 2 h at 4 °C. Dehydrated samples were processed for embedding in epoxy resin (Sigma–Aldrich), and ultrathin sections were analyzed by transmission electron microscopy in a blinded manner (LEO 906E; Oberkochen).

### Statistical analysis

To determine statistical significance between two groups, the unpaired two-tailed Student's *t* test was used. Significance was calculated for at least three biological replicates (n ≥ 3), and *p* values are indicated (∗*p* < 0.05, ∗∗*p* < 0.01, and ∗∗∗*p* < 0.001). For mass spectrometry analysis, the FDR cutoff was set to 0.05. Mean values and SD are given.

## Data availability

The single RNA-Seq dataset was deposited in the Gene Expression Omnibus repository, accession number GSE173204, and the mass spectrometry dataset was deposited in the ProteomeXchange Consortium *via* the PRIDE partner repository with the dataset identifier PXD027109.

## Supporting information

This article contains supporting information.

## Conflict of interest

The authors declare that they have no conflicts of interest with the contents of this article.
