# Supplementary material for: Mitochondrial respiratory chain function promotes extracellular matrix integrity in cartilage
Source: J Biol Chem. 2021 Sep 22;297(4):101224. doi: 10.1016/j.jbc.2021.101224 (PMC8503590; doi:10.1016/j.jbc.2021.101224)
Supplement: Supplemental Figures S1 and S2 [file mmc1.pdf]

Mitochondrial respiratory chain function promotes extracellular matrix integrity in cartilage

Kristina Bubb, Tatjana Holzer, Janica L. Nolte, Marcus Krüger, Richard Wilson, Ursula Schlötzer-Schrehardt, Jürgen Brinckmann, Janine Altmüller, Attila Aszodi, Lutz Fleischhauer, Hauke Clausen-Schaumann, Kristina Probst, Bent Brachvogel

List of supplemental material included:

- Figure S1
- Figure S2

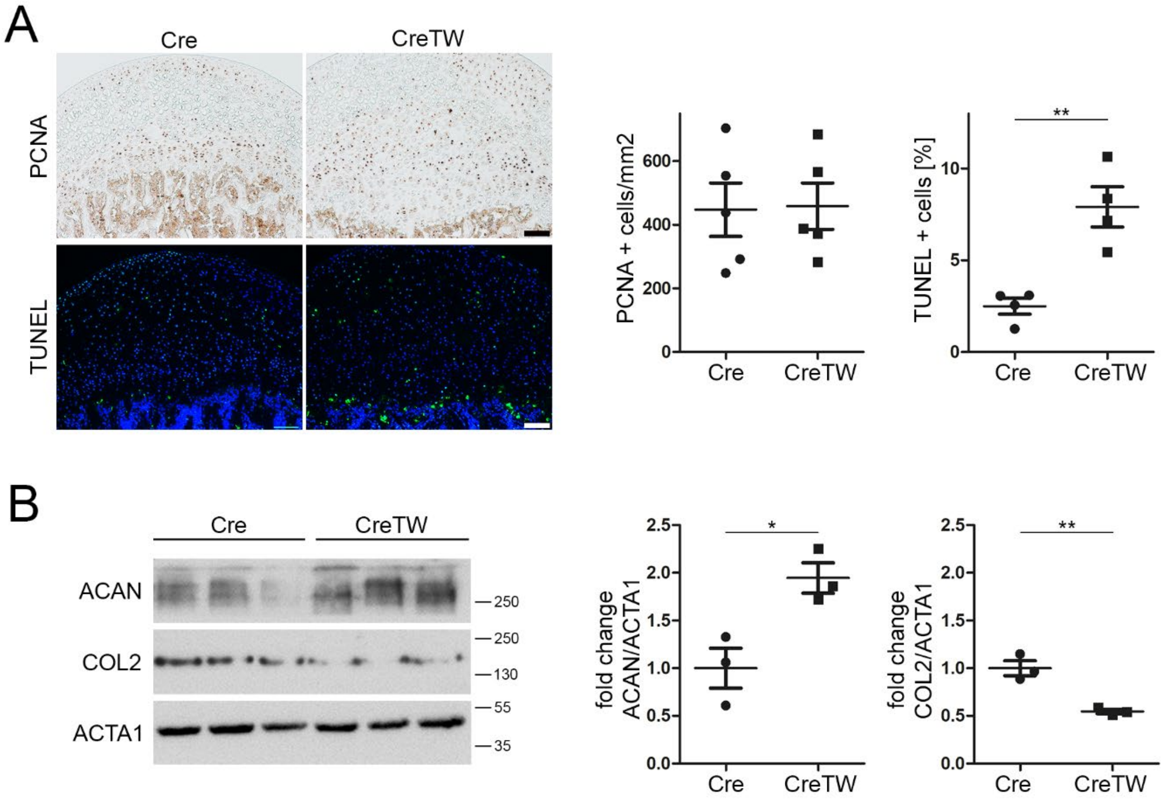

Figure S1 Proliferation, cell death and ECM production. (A) Number of PCNA^{+} and TUNEL^{+} chondrocytes were determined by immunostainings of paraffin sections from PFE cartilage of 1-month-old Cre and CreTW mice. (graphs) The ratio of PCNA- positive chondrocytes per mm^2 cartilage area and the percentage of dying TUNEL positive cells within the total cell population are given. Bars: 100µm. The brightness was adjusted for visualization. (B) Aggrecan (ACAN) and collagen II (COL2) protein levels in cartilage extracts from 1-month-old Cre and CreTW mice were determined by immunoblot. Actin (ACTA1) was used for normalization. (graphs) Fold changes in protein levels between Cre and CreTW are shown. (mean ± SD. *p<0.05, **p<0.01).

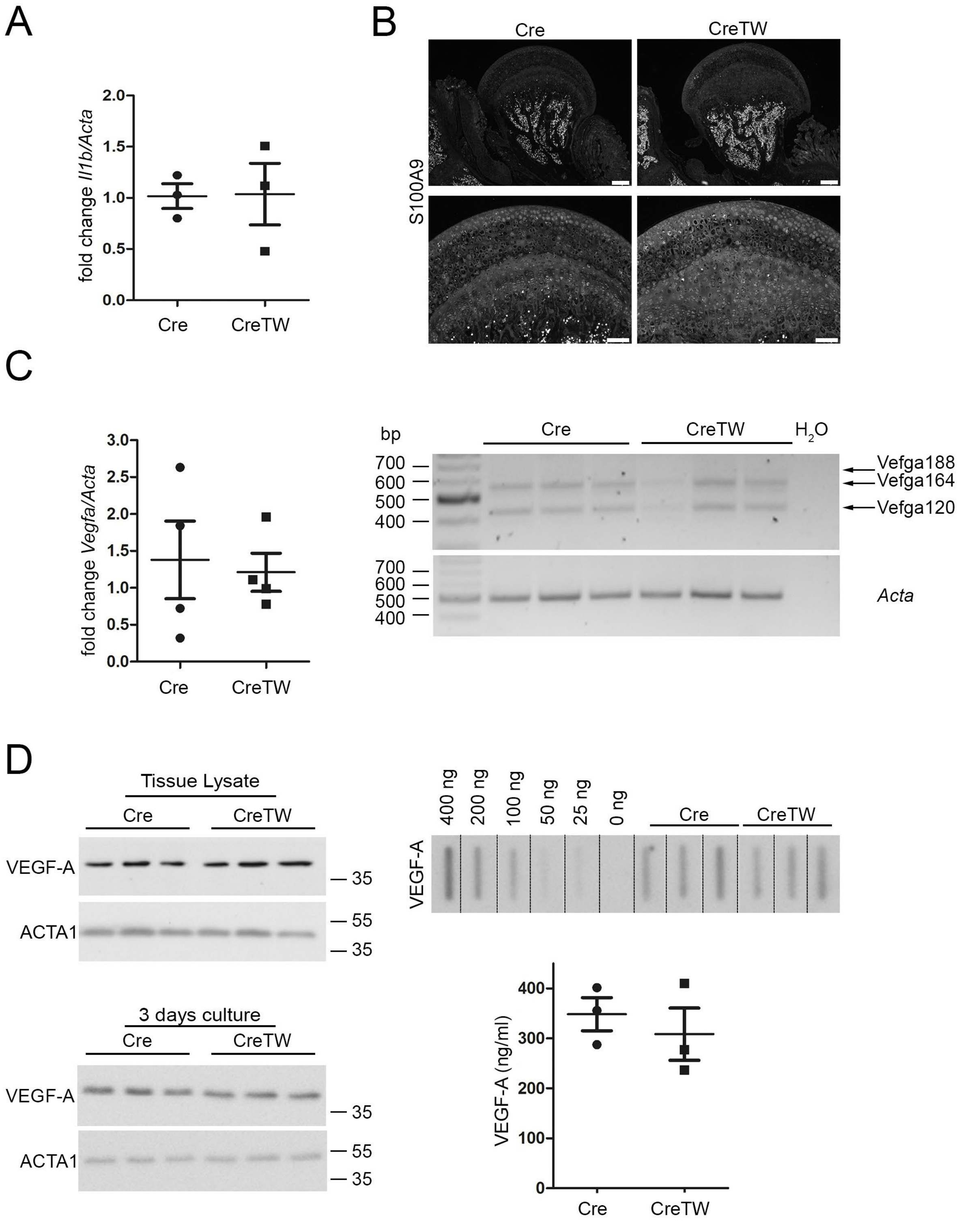
A
fold change Il1b/Acta
2.0
1.5
1.0
0.5
0.0
Cre
CreTW
B
Cre
CreTW
S100A9
C
fold change Vegfa/Acta
3.0
2.5
2.0
1.5
1.0
0.5
0.0
Cre
CreTW
bp
Cre
CreTW
H2O
700
600
500
400
Vefga188
Vefga164
Vefga120
Acta
D
Tissue Lysate
Cre
CreTW
VEGF-A
ACTA1
35
55
35
3 days culture
Cre
CreTW
VEGF-A
ACTA1
35
55
35
400 ng
200 ng
100 ng
50 ng
25 ng
0 ng
Cre
CreTW
VEGF-A
VEGF-A (ng/ml)
400
300
200
100
0
Cre
CreTW

Figure S2 Inflammation and angiogenesis. (A) Quantitative real time PCR analysis of interleukin 1 beta (*Il1b*) expression in PFE cartilage from 1-month-old Cre and CreTW. Fold changes are shown. Values were normalized to actin (*Acta)* (n=3). (B) S100A9 distribution in paraffin sections of 1-month-old Cre and CreTW mice was determined by immunofluorescence microscopy. Bars: 200µm (overview), 100µm (close up) (n=3). (C) Quantitative real time PCR analysis was used to define *Vegfa* relative expression levels of VEGFA (graph; n=3). Characterization of *Vegfa* isoform (*Vegfa188, Vegfa164, Vegfa120*) expression by semiquantitative PCR (n=3). (D) Immunoblot analysis of PFE tissue lysates, chondrocyte cell culture lysates and slot blot analysis of the supernatant of cultured chondrocytes was used to determine VEGFA protein levels. Quantification of the VEGFA concentration in supernatant is given (graph; n=3).
